# Human induced-pluripotent stem cell-derived hepatocyte-like cells as an *in vitro* model of human hepatitis B virus infection

**DOI:** 10.1038/srep45698

**Published:** 2017-04-04

**Authors:** Fuminori Sakurai, Seiji Mitani, Tatsuro Yamamoto, Kazuo Takayama, Masashi Tachibana, Koichi Watashi, Takaji Wakita, Sayuki Iijima, Yasuhito Tanaka, Hiroyuki Mizuguchi

**Affiliations:** 1 Laboratory of Biochemistry and Molecular Biology, Graduate School of Pharmaceutical Sciences, Osaka University, Osaka, Japan; 2 Laboratory of Regulatory Sciences for Oligonucleotide Therapeutics, Clinical Drug Development Unit, Graduate School of Pharmaceutical Sciences, Osaka University, Osaka, Japan; 3 iPS Cell-Based Research Project on Hepatic Toxicity and Metabolism, Graduate School of Pharmaceutical Sciences, Osaka University, Osaka, Japan; 4 Laboratory of Hepatocyte Regulation, National Institute of Biomedical Innovation, Health and Nutrition, Japan; 5 Department of Virology 2, National Institute of Infectious Diseases, Japan; 6 Department of Virology & Liver unit, Nagoya City University Graduate School of Medical Sciences, Nagoya, Japan; 7 Global Center for Medical Engineering and Informatics, Osaka University, Osaka, Japan; 8 Graduate School of Medicine, Osaka University, Osaka, Japan

## Abstract

In order to understand the life cycle of hepatitis B virus (HBV) and to develop efficient anti-HBV drugs, a useful *in vitro* cell culture system which allows HBV infection and recapitulates virus-host interactions is essential; however, pre-existing *in vitro* HBV infection models are often problematic. Here, we examined the potential of human induced-pluripotent stem (iPS) cell-derived hepatocyte-like cells (iPS-HLCs) as an *in vitro* HBV infection model. Expression levels of several genes involved in HBV infection, including the sodium taurocholate cotransporting polypeptide (NTCP) gene, were gradually elevated as the differentiation status of human iPS cells proceeded to iPS-HLCs. The mRNA levels of these genes were comparable between primary human hepatocytes (PHHs) and iPS-HLCs. Following inoculation with HBV, we found significant production of HBV proteins and viral RNAs in iPS-HLCs. The three major forms of the HBV genome were detected in iPS-HLCs by Southern blotting analysis. Anti-HBV agents entecavir and Myrcludex-B, which are a nucleoside analogue reverse transcriptase inhibitor and a synthetic pre-S1 peptide, respectively, significantly inhibited HBV infection in iPS-HLCs. These data demonstrate that iPS-HLCs can be used as a promising *in vitro* HBV infection model.

Hepatitis B virus (HBV) is an enveloped DNA virus that exhibits persistent infection in the human liver. There are almost 350 million people chronically infected with HBV worldwide^1^. Chronic infection with HBV in the liver causes severe liver diseases, including liver cirrhosis and cancer^2^,^3^. HBV thus remains one of the most serious of public health concerns. While effective HBV vaccines and several anti-HBV agents, including nucleoside analogues, are currently available, there are several problems with the present treatment regimens for chronic HBV infection, including the emergence of drug-resistant HBV^4^,^5^. The development of strategies and agents which can efficiently eliminate HBV from the liver without apparent side effects is eagerly anticipated.

An *in vitro* HBV infection model which mimics HBV infection in the human liver is crucial not only to clarify the HBV life cycle but also for the development of anti-HBV agents. HBV shows a highly narrow host range and a strong tropism for hepatocytes. Currently, several cell lines, including a human hepatoma HepaRG cell line and primary human hepatocytes (PHHs), are used as models of HBV infection *in vitro*;^6^,^7^ however, several drawbacks have been found in these *in vitro* HBV infection models. In the case of HepaRG cells, for example, it is impossible to evaluate the effects of genetic background on HBV infection using HepaRG cells. PHHs have restricted availability although PHHs isolated from young children can proliferate in humanized chimera mice^8^,^9^. There is thus urgent need of a novel *in vitro* HBV infection model.

Recently, human hepatocyte-like cells differentiated from human embryonic stem (ES) cells and induced-pluripotent stem (iPS) cells have gained much attention not only due to their promise for regenerative medicines, but also due to their potential for modeling drug metabolism and pathogen infection *in vitro*^10^,^11^,^12^,^13^,^14^. For example, human hepatocyte-like cells differentiated from these stem cells can be stably supplied thanks to the indefinite proliferation potential of ES and iPS cells. Influences on the genetic background of the cells can be evaluated because iPS cells are established from various types of somatic cells. In addition, genome editing technology makes gene knockout and gene replacement possible in human ES and iPS cells^15^. Our group previously developed an efficient protocol for differentiating human iPS cells to hepatocyte-like cells^11^,^13^,^16^,^17^,^18^,^19^. The resulting, iPS-derived hepatocyte-like cells (iPS-HLCs) exhibit gene expression profiles highly similar to PHHs, and may be useful not only to predict interindividual differences in drug metabolism capacity and drug responses but also to allow replication of the hepatitis C virus replicon genome^11^,^13^,^16^,^20^. We thus considered that iPS-HCLs are promising as an alternative *in vitro* model of infection by hepatotropic pathogens, including HBV.

In this study, we examined the potential of iPS-HLCs as an *in vitro* infection model of HBV. iPS-HLCs expressed HBV infection-related cellular factors at levels similar to PHHs. iPS-HLCs were successfully infected by HBV, leading to production of HBV antigens and HBV-derived RNAs. These data indicate that HBV would be a promising *in vitro* HBV infection model.

## Results

### Expression levels of HBV infection-related genes in iPS-HLCs

**Figure 1:**
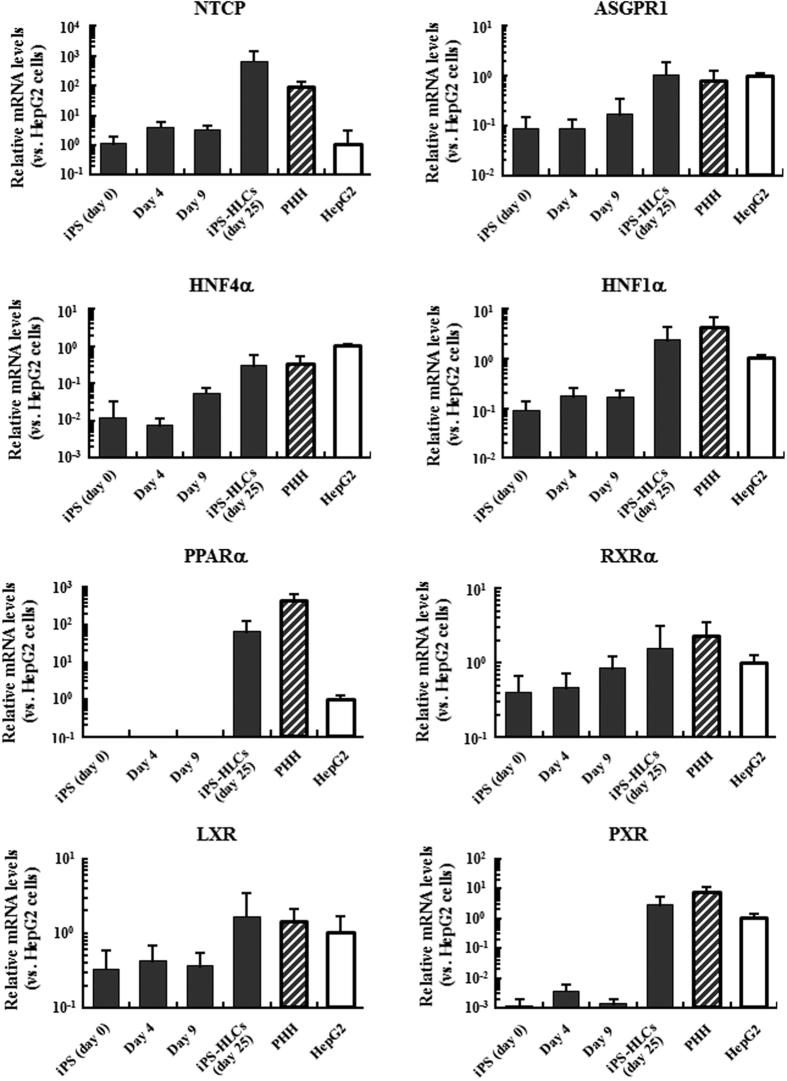
The mRNA levels of genes involved in HBV infection in iPS-HLSs. The mRNA levels in the cells were determined by real-time RT-PCR analysis. The ratios of target genes to GAPDH levels were determined. The data on HepG2 cells was normalized to 1. The data are presented as the mean ± S.D. (n = 4).

In order to examine the expression levels of HBV infection-related genes, including HBV receptor genes, in human iPS cells and iPS cell-derived differentiated cells, including iPS-HLCs, real-time RT-PCR analysis was performed. The expression levels of transcriptional factors and nuclear receptors that were demonstrated to be important for both hepatic functions and HBV infection *via* the regulation of HBV gene expression including hepatocyte nuclear factor 4α (HNF4α) and retinoid X receptor α (RXRα)^21^,^22^,^23^,^24^,^25^ gradually increased as the differentiation of iPS cells to iPS-HCLs proceeded (Fig. 1). The mRNA levels of these genes in iPS-HLCs were similar to those in PHHs. Although almost undetectable or negligible levels of mRNA of peroxisome proliferator-activated receptor α (PPARα) and pregnane X receptor (PXR) were found in undifferentiated human iPS cells, and differentiated iPS cells on culture days 4 and 9, the mRNA levels of these genes in iPS-HLCs were comparable to or slightly lower than those in PHHs. We previously demonstrated that, judging from the gene expression profile data, the differentiation stages of the iPS cells on culture days 9 and 14 correspond to definitive endoderm cells and hepatoblast-like cells^16^. The mRNA levels of asialoglycoprotein receptor-1 (ASGPR1) and sodium-taurocholate cotransporting peptide (NTCP), which were transmembrane proteins involved in HBV infection^26^,^27^, were also gradually elevated depending on the differentiation status and comparable to or slightly higher than those in PHHs. These results indicate that iPS-HLCs expressed several genes crucial for HBV infection at levels comparable to those in PHHs.

### Expression of HBV genes in iPS-HLCs following adenovirus vector-mediated introduction of the HBV genome

**Figure 2:**
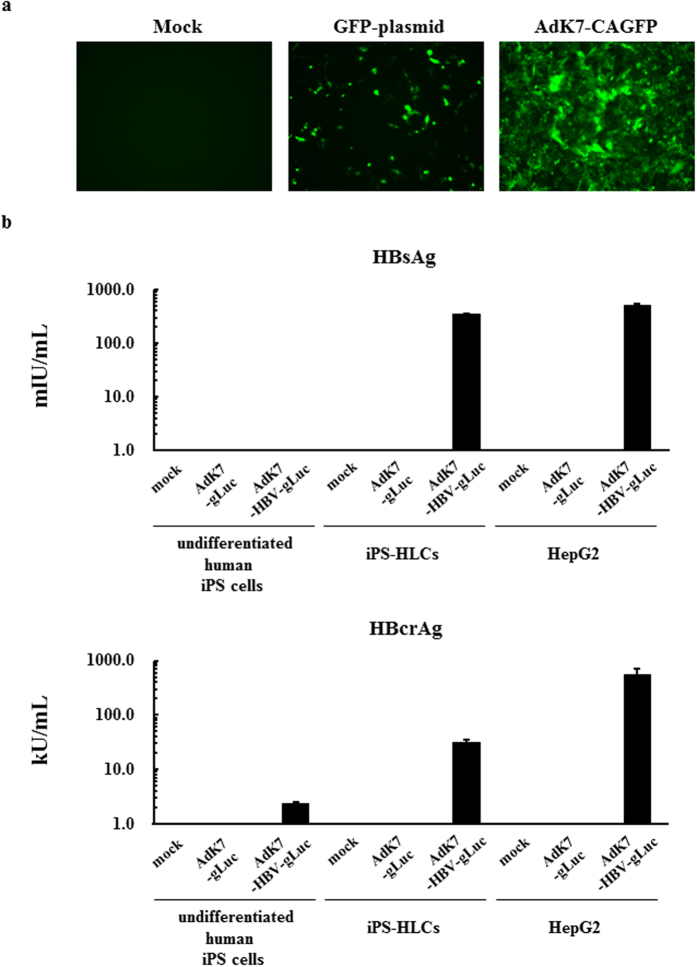
HBV protein production following Ad vector-mediated introduction of the HBV genome in iPS-HLCs. (**a**) GFP expression levels following introduction of the GFP gene in iPS-HLCs. iPS-HLCs were transfected with pHMCA5-GFP using Lipofectamine2000 for 4 h or transduced with AdK7-CAGFP at 300 VP/cell for 2 h. GFP expression levels were evaluated 72-h after introduction of the GFP gene. (**b**) HBsAg and HBcrAg levels in the culture supernatants following introduction of the HBV genome. Human undifferentiated iPS cells, iPS-HLCs, and HepG2 cells were transduced with an Ad vector containing the HBV genome for 2 h. Following a total 72-h incubation, the levels of HBsAg and HBcrAg in the culture supernatants were determined by CLEIA. The data are presented as the mean ± S.D. (n = 3).

Next, in order to examine whether HBV genes were efficiently expressed in iPS-HLCs, the HBV genome was introduced in iPS-HLCs by a fiber-modified Ad vector containing a polylysine peptide in the C-terminal region of the fiber knob^28^. AdK7-CAGFP efficiently transduced more than 90% of iPS-HLCs, although transfection with a GFP-expressing plasmid using a transfection reagent resulted in less than 5% GFP-positive cells (Fig. 2a). Following introduction of the HBV genome, iPS-HLCs produced significant levels of HBsAg and HBcrAg at levels lower than those in HepG2 cells, but much higher than those in undifferentiated human iPS cells (Fig. 2b). HBsAg and HBcrAg levels in the culture supernatants of undifferentiated human iPS cells were slightly above the background levels following introduction of the HBV genome, although the levels of Ad vector genome were comparable between undifferentiated human iPS cells and iPS-HLCs (data not shown). Previous studies demonstrated that the expression of HBV genes following transfection with the HBV genome occurred more efficiently in the hepatocyte cell lines than the non-hepatic cell lines and that hepatocyte-specific transcriptional factors were involved in the transcription of HBV genes^21^,^22^,^29^. These results indicate that iPS-HLCs allowed efficient expression of the HBV genome following its introduction.

### Inoculation with HBV in iPS-HLCs

**Figure 3:**
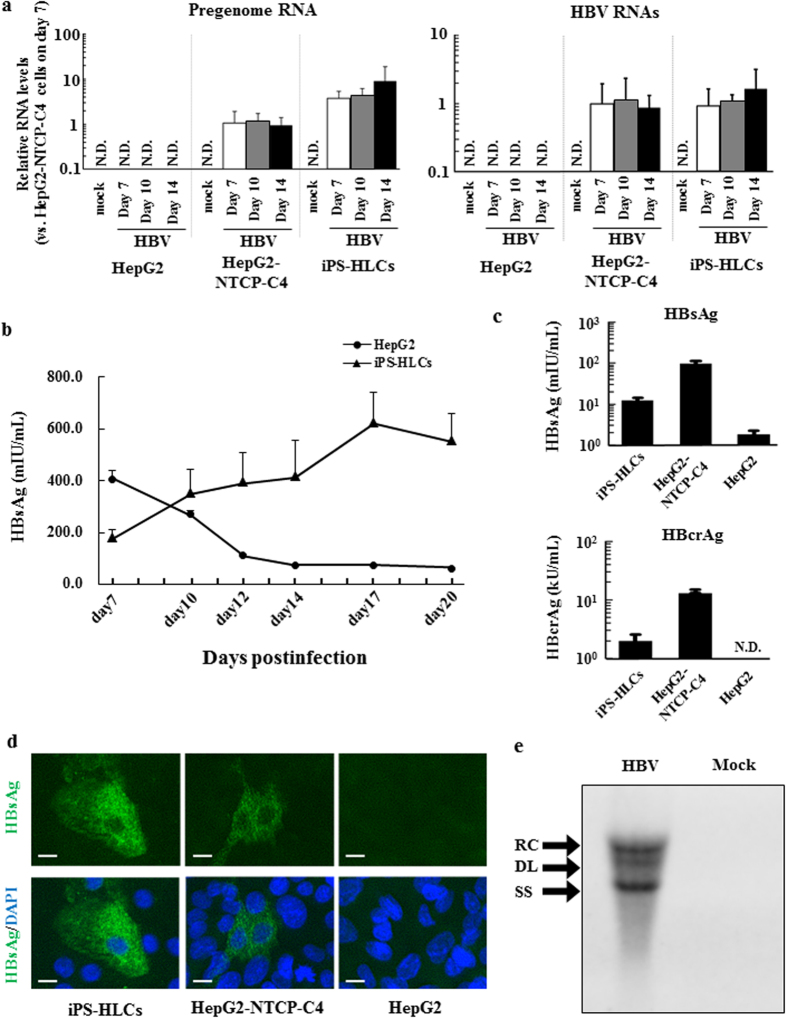
HBV infection levels in iPS-HLCs. iPS-HLCs were inoculated with HBV (genotype D) at 5000 GEq/cell for 24 h. The cells were washed out on days 1, 2, 3, 5, and 7. (**a**) Levels of HBV RNAs and pgRNA in iPS-HLCs following inoculation with HBV. Total RNA was isolated from the cells 7, 10, and 14 days after inoculation. The ratios of pgRNA and HBV RNAs to GAPDH levels were determined. (**b**) Time-course of HBV protein levels in the culture supernatants following inoculation. (**c**) HBV protein levels in the culture supernatants after a total 10 day-culture. HBV protein levels were determined by CLEIA. (**d**) HBsAg expression in iPS-HLCs. Immunostaining analysis of HBsAg (green) was performed 10 days after inoculation. Cell nuclei were counterstained with DAPI (blue). Scale bars indicate 10 μm. Representative images of three independent experiments are shown. (**e**) Southern blotting analysis of the HBV genome in iPS-HLCs. Representative images of two independent experiments are shown. The data are presented as the mean ± S.D. (n = 3). N.D.; not detected.

In order to examine whether iPS-HLCs allow HBV infection, iPS-HLCs were inoculated with HBV for 24 h and subsequently cultured in normal cultured medium. The HBV inoculum used in this experiment was genotype D, and was derived from the culture supernatant of HepG2.2.15.7 cells^30^, which are a HepG2.2.15 subclone producing a high titer of HBV. HBV pregenome (pg) RNA and total HBV RNAs were detected in iPS-HLCs following inoculation (Fig. 3a). The copy numbers of HBV pgRNA and total RNAs were comparable to or slightly higher than those in HepG2-NTCP-C4 cells, although the copy numbers of these RNAs were almost constant during culture in both iPS-HLCs and HepG2-NTCP-C4 cells. Detectable levels of HBV pgRNA and total RNAs were not found in undifferentiated human iPS cells after inoculation with HBV for 24 h, followed by 5-day culture (Fig. S2). HepG2 cells, which express undetectable levels of NTCP, did not produce HBV pgRNA or HBV RNAs in the cells.

Next, HBV protein expression in iPS-HLCs following inoculation with HBV was examined. HBsAg levels in the culture supernatants gradually increased after infection up to day 17 (Fig. 3b). iPS-HLCs produced almost 10-fold lower levels of HBsAg and HBcrAg in the culture supernatants than HepG2-NTCP-C4 cells on day 10 after inoculation (Fig. 3c). HBV-derived RNA and HBV proteins were also efficiently expressed in the iPS-HLCs differentiated from the other human iPS cell lines (Fig. S3). Immunostaining analysis of iPS-HLCs also demonstrated that HBsAg was expressed in both iPS-HLCs and HepG2-NTCP-C4 cells, but not HepG2 cells, although the HBsAg expression levels in iPS-HLCs were lower than those in HepG2-NTCP-C4 cells (Fig. 3d). The numbers of HBsAg-positive iPS-HLCs did not increase during culture (data not shown). Moreover, in order to examine whether HBV genome replication occurs in iPS-HLCs, the HBV genome was extracted from cytoplasmic core particles and subjected to southern blotting analysis. Not only the relaxed circular^31^ form but also the single-stranded linear form, which is a replication intermediate, was detected in iPS-HLCs (Fig. 3e). The data described above indicate that iPS-HLCs permit HBV infection.

### Suppression of HBV infection in iPS-HLCs by anti-HBV agents

**Figure 4:**
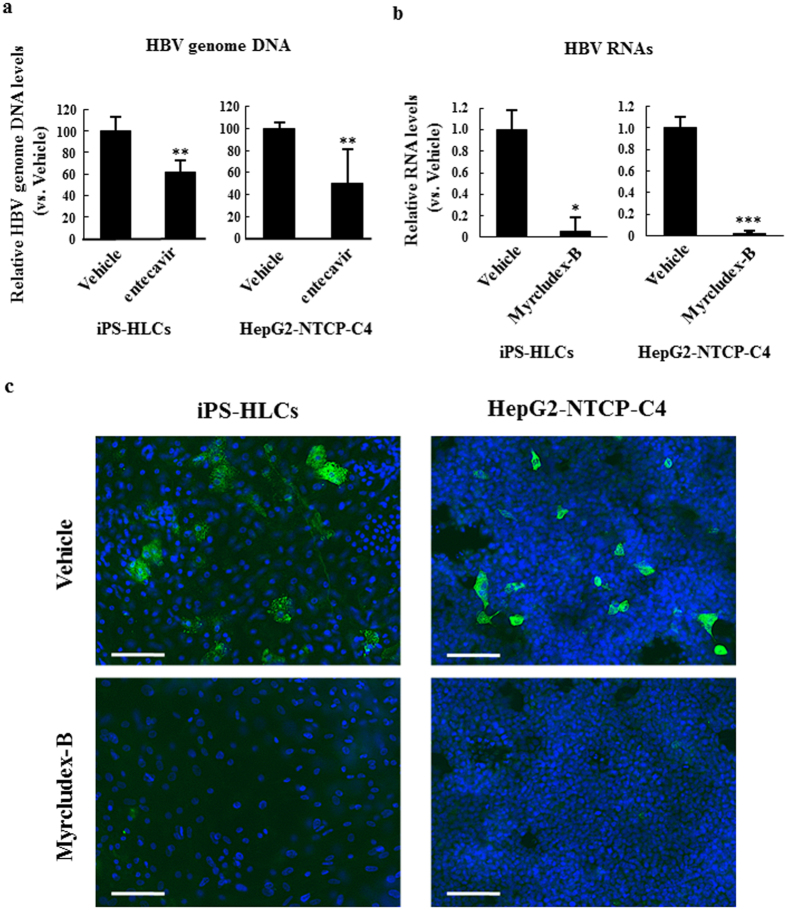
Inhibition of HBV infection in iPS-HLCs by entecavir and Myrcludex-B. (**a**) Copy numbers of HBV genome in the culture supernatants. Twenty-four hours after inoculation with HBV, iPS-HLCs were treated with 1 μM entecavir. The HBV genome copy numbers were determined by real-time PCR analysis at 10 days after inoculation. (**b,c**) Expression levels of HBV RNAs and HBsAg in iPS-HLCs pre-treated with Myrcludex-B. iPS-HLCs were pre-treated with Myrcludex-B at 50 nM for 3 h. iPS-HLCs were subsequently inoculated with HBV (genotype D) at 5000 GEq/cell for 24 h. Expression levels of (**b**) HBV RNAs and (**c**) HBsAg were determined 10 days after inoculation. HBV RNA levels in iPS-HLCs were determined by real-time RT-PCR analysis. The ratios of HBV RNAs to GAPDH levels were determined. The data are presented as the mean ± S.D. (n = 3). *p < 0.05, **p < 0.01, ***p < 0.001 (compared with vehicle). HBsAg expression (green) in iPS-HLCs was evaluated by immunostaining using anti-HBsAg antibody. Cells were counterstained by DAPI (blue). Scale bars indicate 200 μm. Representative images of two independent experiments are shown.

Next, in order to examine the potential of iPS-HLCs as an *in vitro* model for drug screening of anti-HBV agents, entecavir and Myrcludex-B was tested in iPS-HLCs. Entecavir is an oral nucleotide analogue drug for the treatment of chronic HBV infection that act by inhibiting reverse transcription^32^. Myrcludex-B is a lipopeptide consisting of amino acid residues 2–48 of the pre-S1 region of HBV, and is known to block HBV entry^33^,^34^,^35^. Entecavir significantly reduced the HBV genome levels in the culture media of iPS-HLCs by approximately 40% (Fig. 4a). Myrcludex-B largely reduced the mRNA levels of pgRNA and HBV RNAs in iPS-HCLs (Fig. 4b). Moreover, the numbers of HBsAg-positive cells were also significantly reduced by Myrcludex-B (Fig. 4c). Apparent toxicities were not found in iPS-HLCs following incubation with Myrcludex-B (data not shown). These results indicate that iPS-HLCs are a promising *in vitro* model for drug screening of anti-HBV agents.

## Discussion

Various *in vitro* models of HBV infection model, including HepaRG cells and primary human and Tupaia hepatocytes, have been reported^6^,^36^,^37^. Compared with these previously reported *in vitro* HBV infection models, iPS-HLCs possess several advantages. First, human iPS cells can proliferate infinitely, which makes a stable supply possible. In addition, iPS-HLCs are much less problematic ethically than PHHs. Second, human iPS cells can be established from somatic cells with various genetic backgrounds. The influence of differences in the genetic backgrounds of host cells on HBV infection levels can be evaluated using iPS-HLCs. Several studies have demonstrated the association of single nucleotide polymorphisms in several genes with HBV infection^38^,^39^,^40^. In addition, we previously demonstrated that the cytochrome P450 (CYP) metabolism capacity and drug responsiveness of hepatocyte-like cells differentiated from PHH-derived iPS cells (PHH-iPS-HLCs) were highly correlated with those of PHHs, suggesting that the iPS-HLCs retained donor-specific CYP metabolism capacity and drug responsiveness^13^. Not only anti-HBV infection activity of anti-HBV agents but also hepatic metabolism of anti-HBV agents can be simultaneously evaluated in iPS-HLCs. Third, HBV-induced innate immune responses can be evaluated in iPS-HLCs. HBV induces innate immune responses *via* several types of pattern recognition receptors, including retinoic acid-inducible gene-I (RIG-I) and toll-like receptor (TLR) 3^41^,^42^. HBV-induced innate immunity is a crucial host response for elimination of HBV invaded into host cells^43^,^44^; however, it is difficult to evaluate innate immune responses in most of human hepatoma cell lines, including HepG2 cells and Huh-7 cells. By contrast, Shlomai *et al*. demonstrated HBV-induced innate immune responses in iPS cell-derived hepatocyte-like cells^45^. These advantages of iPS-HLCs make iPS-HLCs highly promising as an *in vitro* HBV infection model.

On the other hand, HBV proliferation levels in iPS-HLCs seems to be lower than those in PHHs. HBV antigen-positive cells have been shown to increase in PHHs following inoculation^9^,^45^ while in the present study HBV antigen-positive cells did not increase in iPS-HLCs following inoculation (data not shown). These results indicate that HBV titers produced in the culture supernatants of iPS-HLCs were much lower than those of PHHs. Further maturation of iPS-HLCs might be required for high levels of HBV infection. Three-dimensional (3D) culture of iPS-HLCs is a promising approach for further maturation of iPS-HLCs. Several groups, including ours, also demonstrated that expression of hepatocyte-specific genes in iPS-HLCs was significantly elevated by 3D-culture^11^,^46^,^47^,^48^.

In this study, HBsAg and HBcrAg levels in the culture supernatants of iPS-HLCs were significantly lower than those of HepG2-NTCP-C4 cells after HBV inoculation, while pgRNA and HBV RNA levels in iPS-HLCs were higher than those in HepG2-NTCP-C4 cells. This was attributed to the differences in the cell numbers and total RNA levels recovered from the cells between iPS-HLCs and HepG2-NTCP-C4 cells. That is, although HepG2-NTCP-C4 cells actively proliferated, iPS-HCLs did not, and an approximately 5-fold higher levels of total RNA was recovered from HepG2-NTCP-C4 cells compared with iPS-HLCs.

As described above, the iPS-HLCs used in this study are less differentiated than adult PHHs. iPS-HLCs express both adult hepatocyte-specific and fetal hepatocyte-specific genes. Baxter *et al*. also reported that hepatocyte-like cells derived from human ES and iPS cells exhibited a phenotype similar to human fetal hepatocytes^49^. Studies of HBV infection in iPS-HLCs might provide clues toward the elucidation of HBV infection profiles in fetal hepatocytes. HBV infection in fetal hepatocytes *via* maternal-fetal transmission is the major pathway of HBV infection.

Hepatocyte-like cells differentiated from human ES or iPS cells have been used as an *in vitro* infection model for other pathogens, including hepatitis C virus (HCV) and Plasmodium^20^,^31^,^50^. Replication of these pathogens and innate immune responses were found following inoculation with these pathogens to iPS-HLCs. Now various types of cells, including neuron and muscle cells, can be efficiently differentiated from human ES and iPS cells. Various types of differentiated cells derived from human ES and iPS cells are also promising as an *in vitro* infection model for various pathogens.

While this study was underway, Shlomai *et al*. reported that hepatocyte-like cells differentiated from human iPS cells can support HBV infection^45^. In their study, efficient HBsAg production was observed in the culture medium after infection; however, the HBsAg levels gradually declined to the background levels. In the present study, on the other hand, the HBsAg levels in the culture medium gradually increased up to 17 days after infection in iPS-HLCs of this study, indicating that iPS-HLCs can support long-term HBV infection.

In conclusion, we demonstrated that HBV can infect iPS-HLCs, and therefore that iPS-HLCs are a promising *in vitro* HBV infection model. iPS-HLCs would be a highly crucial tool not only for the elucidation of HBV infection mechanisms but also for the development of anti-HBV agents. In the future, it will be important to examine whether the influence of the genetic background of host cells on HBV infection levels can be evaluated using iPS-HLCs. This project is now underway.

## Materials and Methods

### Cell culture

HepG2 cells (a human hepatocellular carcinoma cell line, RCB1648, obtained from the JCRB Cell Bank), and those stably expressing NTCP (HepG2-hNTCP-C4)^33^ were cultured in Dulbecco’s modified Eagle medium (DMEM) containing 10% fetal bovine serum (FBS) and antibiotics. HepG2.2.15.7 cells^30^, which are a HepG2.2.15 clone producing a higher level of HBV, were also cultured in DMEM/F-12-Glutamax (Invitrogen, Carlsbad, CA) supplemented with 10% FBS, 100 U/ml penicillin, 100 μg/ml streptomycin, 5 μg/ml insulin, and 10 mM HEPES. All cultures were maintained at 37 °C in a humidified atmosphere containing 5% CO2. The human iPS cell line, Dotcom (JCRB1327, obtained from the JCRB Cell Bank), were maintained as previously described^17^,^18^.

### Differentiation of human iPS cells to the definitive endoderm, hepatoblast-like cells, and hepatocyte-like cells

iPS-HLCs were differentiated from the human iPS cell line, Dotcom, as previously described^13^. For the definitive endoderm differentiation, the human iPS cells were cultured for 4 days in L-Wnt3A-expressing cell (ATCC, CRL2647)-conditioned RPMI1640 medium, which contains 100 ng/ml activin A (R&D Systems, Minneapolis, MN), 4 mM L-glutamine, 0.2% FBS, and 1xB27 Supplement Minus Vitamin A (Life Technologies, Carlsbad, CA). For the induction of hepatoblast-like cells (HBCs), the definitive endoderm cells were cultured for 5 days in RPMI1640 medium containing 30 ng/ml bone morphogenetic protein 4 (BMP4) (R&D Systems), 20 ng/ml fibroblast growth factor-4 (FGF4) (R&D Systems), 4 mM L-glutamine, and 1xB27 Supplement Minus Vitamin A. For the differentiation to hepatocyte-like cells (HLCs), the HBCs were cultured for 5 days in RPMI1640 medium containing 20 ng/ml hepatocyte growth factor (HGF) (R&D Systems), 4 mM-L-glutamine, and 1xB27 Supplement Minus Vitamin A. Finally, the cells were cultured for 11 days in Hepatocyte Culture Medium (HCM) (Lonza, Basel, Switzerland) with 20 ng/ml oncostatin M.

### Expression analysis of HBV infection-related genes

Total RNA was recovered from PHHs, HepG2 cells, and the human iPS cells at the different differentiation stages using ISOGEN (Nippon Gene, Tokyo, Japan). Total RNA of PHHs was a mixture of that of PHHs from 3 different donors. Cryopreserved PHHs were obtained from CellzDirect (lot; Hu8072) (Durham, NC) and Xenotech (lots HC2-14 and HC10-101) (Lenexa, KS). Total RNA was isolated from PHHs following a 48-h culture after thawing^16^. cDNA was synthesized using 500 ng of total RNA with a Superscript VILO cDNA synthesis kit (Thermo Fisher Scientific, Rockford, IL). Real-time RT-PCR analysis was performed using Fast SYBR Green Master Mix (Thermo Fisher Scientific) and StepOnePlus real-time PCR systems (Thermo Fisher Scientific). The sequences of the primers for NTCP were as follows; 5′-GGGATCTATGATGGGGACCT-3′, and 5′-GATCCCTATGGTGCAAGGAA-3′. The sequences of the other primers used in this study are previously described^16^.

### Preparation of a fiber-modified adenovirus (Ad) vector containing the HBV genome

A fiber-modified adenovirus (Ad) vector containing the HBV genome was prepared by an improved *in vitro* ligation method^51^,^52^. Briefly, the genotype C HBV genome isolated from pHBV/C-AT^53^ was cloned into pHMCMV5^52^, yielding pHMCMV5-HBV. Next, the gaussia luciferase (gLuc) gene isolated from pGLuc-Basic2 (New England Biolabs, Hertfordshire, UK) was cloned into the downstream region of the CMV promoter in pHMCMV5-HBV, creating pHMCMV5-HBV-gLuc. pHMCMV5-gLuc was constructed by insertion of the gLuc gene into pHMCMV5. I-*Ceu*I/PI-*Sce*I-digested pHMCMV5-HBV-gLuc was ligated with I-*Ceu*I/PI-*Sce*I-digested pAdHM41-K7^28^, yielding pAdHM41-K7-HBV-gLuc. pAdHM41-K7 encodes a polylysine peptide (K7) in the C-terminal region of the Ad fiber knob. pAdHM41-K7-HBV-gLuc was digested with *Pac*I to release the recombinant viral genome, and was transfected into 293 cells plated on 60-mm dishes, resulting in AdK7-HBV-gLuc. AdK7-gLuc, which is a control Ad vector expressing gLuc, was similarly prepared using pHMCMV5-gLuc and pAdHM41-K7. A green fluorescence protein (GFP)-expressing Ad vector containing a K7 in the C-terminal region of the Ad fiber knob (AdK7-CAGFP) was similarly constructed using pAdHM41-K7 and pHMCA5-GFP^54^. Schematic diagrams of the Ad vector genome used in this study are shown in Figure S1. Ad vectors were propagated in 293 cells, purified by two rounds of cesium chloride-gradient ultracentrifugation, dialyzed, and stored at −80 °C. The numbers of virus particles (VP) were determined using a spectrophotometric method^55^.

### Ad vector-mediated introduction of the HBV genome

The Ad vector containing the HBV genome (AdK7-gLuc-HBV) was added to the cells at 300 VP/cell. Following a 72-h incubation, HBsAg and HBcrAg levels in the culture supernatants were determined by chemiluminescent enzyme immunoassay (CLEIA) as described below.

### Inoculation with HBV

The HBV mainly used in this study, which is classified as genotype D, was derived from the culture supernatant of HepG2.2.15.7 cells. The culture supernatants of Hep2.2.15.7 cells were recovered and centrifuged for 15 min at 3500 g and cleared through a 0.45 μm filter to remove cell debris, followed by precipitation with 8% polyethylene glycol (PEG) 6000 (Nacalai Tesque, Kyoto, Japan). The precipitates were washed and resuspended with the medium at approximately 100-fold concentration. The HBV DNA copy numbers were determined by real-time PCR analysis. HBV infection was performed as described below. Cells were inoculated with HBV at the indicated genome equivalent (GEq)/cell in the presence of 4% PEG6000. Following a 24-h incubation at 37 °C, the cells were gently washed 5 times with PBS and then cultured using the appropriate culture medium. Cell washing and medium change were performed 2 and 3 days after infection. The culture medium was also replaced with fresh medium at 5, 7, 10, 12, 14, and 17 days after infection.

### Quantification of HBV RNAs and pre-genome RNA

For determination of the copy numbers of HBV RNAs, including HBV pre-genome RNA (pgRNA), cells were inoculated with HBV as described above. Following incubation, total RNA was recovered and real-time RT-PCR analysis was performed using a Superscript VILO cDNA synthesis kit (Thermo Fisher Scientific), Fast SYBR Green Master Mix (Thermo Fisher Scientific), and StepOnePlus Real-time PCR system (Thermo Fisher Scientific). The sequences of the primers were previously described^42^.

### Southern blotting analysis of HBV genomic DNA

Southern blotting analysis was performed as follows. Briefly, iPS-HLCs were inoculated with HBV at 5000 GEq/cell as described above. Ten days after infection, total DNA, including HBV genome DNA, was recovered from the cells using lysis buffer (50 mM Tris-HCl (pH 7.4), 1 mM EDTA, 1% NP-40). Following treatment of the samples with DNase I and RNase A, the nuclei were pelleted by centrifugation at 4 °C and 15,000 rpm for 5 min. The supernatant was adjusted to 6 mM Mg acetate and treated with 200 μg/ml of DNase I and 100 μg/ml of RNase A for 3 h at 37 °C. The reaction was stopped by the addition of EDTA to a final concentration of 10 mM, and then the mixture was incubated for 10 min at 65 °C. Proteins of the sample were digested with 200 μg/ml of proteinase K, 1% sodium dodecyl sulfate, and 100 mM NaCl for 2 h at 55 °C. Nucleic acids were purified by phenol-chloroform (1:1) extraction and ethanol precipitation after the addition of 20 μg of glycogen. Isolated DNA, including the HBV genome, was separated on a 1.0% agarose gel. DNA was transferred to a positively charged nylon membrane (Roche Diagnostics, Germany) and hybridized with an alkaline phosphatase-labeled full-length HBV fragment generated with a DIG High Prime DNA Labeling and Detection Starter Kit II (Roche Diagnostics). The detection was performed with CDP-Star, ready-to-use (Roche Diagnostics). The signals were analyzed by using a LAS-4000 image analyzer (Fuji Photo Film, Japan).

### Chemiluminescent enzyme immunoassay (CLEIA) for HBsAg and HBcrAg

Cells were inoculated with HBV at 5000 GEq/cell as described above. The culture supernatants were collected at the indicated culture days, and protein levels of HBsAg and HBcrAg were determined by CLEIA using LUMIPLUSE G2100 (Fujirebio Inc., Tokyo, Japan). The detection limits of HBsAg and HBcrAg are 5.0 mIU/ml and 1.0 kU/ml, respectively.

### Immunofluorescence analysis of HBV proteins

Following HBV inoculation as described above, cells were washed twice with cold PBS, followed by incubation with 4% paraformaldehyde (PFA)/PBS for 30 min at room temperature for immobilization. Cells were then treated with 0.25% Triton-X100/PBS for 5 min at room temperature following 3 washes with PBS, then blocking with 2% bovine serum albumin (BSA) and 2.5% normal donkey serum (Abcam)/PBS. Cells were incubated with anti-HBsAg goat IgG polyclonal antibody (bs-1557G, 1:1000) (Bioss, Boston, MA) as a primary antibody overnight at 4 °C. After washing, cells were incubated with Alexa Fluor 488-labeled donkey anti-goat IgG (H + L) (A-11055, 1:1000) (Life Technologies) for 1 h. Cells were then stained with 4’,6-diamidino-2-phenylindole (DAPI) for 1 h and observed under a fluorescence microscope.

### Inhibition of HBV infection by Entecavir and Myrcludex-B

Inhibition of HBV infection by Entecavir and Myrcludex-B was performed basically as described previously^33^,^56^. Briefly, HBV was added to the cells at 5000 GEq/cell. Twenty-four hours after addition of HBV, entecavir (final concentration 1 μM) was added to the cells, followed by incubation for 9 days. Copy numbers of the HBV genome in the culture supernatants were determined by real-time PCR analysis following a total 10 day-culture after inoculation with HBV. In the case of Myrcludex-B, cells were incubated with Myrcludex-B for 3 h prior to and 24 h during HBV inoculation. Total RNA was recovered from the cells 10 days after HBV inoculation. HBV RNAs and pgRNA levels were evaluated by real-time RT-PCR analysis as described above. Immunostaining of HBsAg in the cells was also performed 10 days after HBV inoculation as described above.

## Additional Information

**How to cite this article:** Sakurai, F. *et al*. Human induced-pluripotent stem cell-derived hepatocyte-like cells as an *in vitro* model of human hepatitis B virus infection. *Sci. Rep.* **7**, 45698; doi: 10.1038/srep45698 (2017).

**Publisher's note:** Springer Nature remains neutral with regard to jurisdictional claims in published maps and institutional affiliations.

## Supplementary Material

Supplementary Information

## References

[b1] Lee W. M. Hepatitis B virus infection. N Engl J Med 337, 1733–1745 (1997). 9392700 10.1056/NEJM199712113372406

[b2] Neuveut C., Wei Y. & Buendia M. A. Mechanisms of HBV-related hepatocarcinogenesis. J Hepatol 52, 594–604 (2010). 20185200 10.1016/j.jhep.2009.10.033

[b3] Murata M. . Hepatitis B virus X protein shifts human hepatic transforming growth factor (TGF)-beta signaling from tumor suppression to oncogenesis in early chronic hepatitis B. Hepatology 49, 1203–1217 (2009). 19263472 10.1002/hep.22765

[b4] Tenney D. J. . Clinical emergence of entecavir-resistant hepatitis B virus requires additional substitutions in virus already resistant to Lamivudine. Antimicrob Agents Chemother 48, 3498–3507 (2004). 15328117 10.1128/AAC.48.9.3498-3507.2004 PMC514758

[b5] Jardi R. . Hepatitis B virus polymerase variants associated with entecavir drug resistance in treatment-naive patients. J Viral Hepat 14, 835–840 (2007). 18070286 10.1111/j.1365-2893.2007.00877.x

[b6] Gripon P. . Infection of a human hepatoma cell line by hepatitis B virus. Proc Natl Acad Sci USA 99, 15655–15660 (2002). 12432097 10.1073/pnas.232137699 PMC137772

[b7] Gripon P. . Hepatitis B virus infection of adult human hepatocytes cultured in the presence of dimethyl sulfoxide. J Virol 62, 4136–4143 (1988). 3172341 10.1128/jvi.62.11.4136-4143.1988 PMC253845

[b8] Tateno C. . Near completely humanized liver in mice shows human-type metabolic responses to drugs. Am J Pathol 165, 901–912 (2004). 15331414 10.1016/S0002-9440(10)63352-4 PMC1618591

[b9] Ishida Y. . Novel robust *in vitro* hepatitis B virus infection model using fresh human hepatocytes isolated from humanized mice. Am J Pathol 185, 1275–1285 (2015). 25791527 10.1016/j.ajpath.2015.01.028

[b10] Mallanna S. K. & Duncan S. A. Differentiation of hepatocytes from pluripotent stem cells. Curr Protoc Stem Cell Biol 26, Unit 1G 4 (2013). 10.1002/9780470151808.sc01g04s26 PMC3920294 24510789

[b11] Takayama K. . 3D spheroid culture of hESC/hiPSC-derived hepatocyte-like cells for drug toxicity testing. Biomaterials 34, 1781–1789 (2013). 23228427 10.1016/j.biomaterials.2012.11.029

[b12] Dvorak Z. Opportunities and challenges in using human hepatocytes in cytochromes P450 induction assays. Expert Opin Drug Metab Toxicol, 1–6 (2016). 10.1517/17425255.2016.1125881 26612411

[b13] Takayama K. . Prediction of interindividual differences in hepatic functions and drug sensitivity by using human iPS-derived hepatocytes. Proc Natl Acad Sci USA 111, 16772–16777 (2014). 25385620 10.1073/pnas.1413481111 PMC4250156

[b14] Hirschi K. K., Li S. & Roy K. Induced pluripotent stem cells for regenerative medicine. Annu Rev Biomed Eng 16, 277–294 (2014). 24905879 10.1146/annurev-bioeng-071813-105108 PMC4287204

[b15] Li M., Suzuki K., Kim N. Y., Liu G. H. & Izpisua Belmonte J. C. A cut above the rest: targeted genome editing technologies in human pluripotent stem cells. J Biol Chem 289, 4594–4599 (2014). 24362028 10.1074/jbc.R113.488247 PMC3931021

[b16] Takayama K. . Generation of metabolically functioning hepatocytes from human pluripotent stem cells by FOXA2 and HNF1alpha transduction. J Hepatol 57, 628–636 (2012). 22659344 10.1016/j.jhep.2012.04.038

[b17] Takayama K. . Efficient generation of functional hepatocytes from human embryonic stem cells and induced pluripotent stem cells by HNF4alpha transduction. Mol Ther 20, 127–137 (2012). 10.1038/mt.2011.234 PMC3255576 22068426

[b18] Takayama K. . Efficient and directive generation of two distinct endoderm lineages from human ESCs and iPSCs by differentiation stage-specific SOX17 transduction. PLoS One 6, e21780 (2011). 21760905 10.1371/journal.pone.0021780 PMC3131299

[b19] Inamura M. . Efficient generation of hepatoblasts from human ES cells and iPS cells by transient overexpression of homeobox gene HEX. Mol Ther 19, 400–407 (2011). 21102561 10.1038/mt.2010.241 PMC3034848

[b20] Yoshida T. . Use of human hepatocyte-like cells derived from induced pluripotent stem cells as a model for hepatocytes in hepatitis C virus infection. Biochem Biophys Res Commun 416, 119–124 (2011). 22093821 10.1016/j.bbrc.2011.11.007

[b21] Tang H. & McLachlan A. Transcriptional regulation of hepatitis B virus by nuclear hormone receptors is a critical determinant of viral tropism. Proc Natl Acad Sci USA 98, 1841–1846 (2001). 11172038 10.1073/pnas.041479698 PMC29344

[b22] Yu X. & Mertz J. E. Distinct modes of regulation of transcription of hepatitis B virus by the nuclear receptors HNF4alpha and COUP-TF1. J Virol 77, 2489–2499 (2003). 12551987 10.1128/JVI.77.4.2489-2499.2003 PMC141100

[b23] Quasdorff M. . A concerted action of HNF4alpha and HNF1alpha links hepatitis B virus replication to hepatocyte differentiation. Cell Microbiol 10, 1478–1490 (2008). 18346225 10.1111/j.1462-5822.2008.01141.x

[b24] Huan B. & Siddiqui A. Retinoid X receptor RXR alpha binds to and trans-activates the hepatitis B virus enhancer. Proc Natl Acad Sci USA 89, 9059–9063 (1992). 1329088 10.1073/pnas.89.19.9059 PMC50064

[b25] Hu W. . MicroRNA-141 represses HBV replication by targeting PPARA. PLoS One 7, e34165 (2012). 22479552 10.1371/journal.pone.0034165 PMC3316618

[b26] Yan H. . Sodium taurocholate cotransporting polypeptide is a functional receptor for human hepatitis B and D virus. Elife 1, e00049 (2012). 23150796 10.7554/eLife.00049 PMC3485615

[b27] Treichel U. Meyer, zum Buschenfelde K. H., Stockert R. J., Poralla T. & Gerken G. The asialoglycoprotein receptor mediates hepatic binding and uptake of natural hepatitis B virus particles derived from viraemic carriers. J Gen Virol 75 (Pt 11), 3021–3029 (1994). 7964611 10.1099/0022-1317-75-11-3021

[b28] Koizumi N., Mizuguchi H., Utoguchi N., Watanabe Y. & Hayakawa T. Generation of fiber-modified adenovirus vectors containing heterologous peptides in both the HI loop and C terminus of the fiber knob. J Gene Med 5, 267–276 (2003). 12692861 10.1002/jgm.348

[b29] Shaul Y., Rutter W. J. & Laub O. A human hepatitis B viral enhancer element. EMBO J 4, 427–430 (1985). 3926485 10.1002/j.1460-2075.1985.tb03646.x PMC554203

[b30] Yamashita A. . Identification of Antiviral Agents Targeting Hepatitis B Virus Promoter from Extracts of Indonesian Marine Organisms by a Novel Cell-Based Screening Assay. Mar Drugs 13, 6759–6773 (2015). 26561821 10.3390/md13116759 PMC4663552

[b31] Ng S. . Human iPSC-derived hepatocyte-like cells support Plasmodium liver-stage infection *in vitro*. Stem Cell Reports 4, 348–359 (2015). 25660406 10.1016/j.stemcr.2015.01.002 PMC4375936

[b32] Langley D. R. . Inhibition of hepatitis B virus polymerase by entecavir. J Virol 81, 3992–4001 (2007). 17267485 10.1128/JVI.02395-06 PMC1866160

[b33] Iwamoto M. . Evaluation and identification of hepatitis B virus entry inhibitors using HepG2 cells overexpressing a membrane transporter NTCP. Biochem Biophys Res Commun 443, 808–813 (2014). 24342612 10.1016/j.bbrc.2013.12.052

[b34] Gripon P., Cannie I. & Urban S. Efficient inhibition of hepatitis B virus infection by acylated peptides derived from the large viral surface protein. J Virol 79, 1613–1622 (2005). 15650187 10.1128/JVI.79.3.1613-1622.2005 PMC544121

[b35] Schulze A., Schieck A., Ni Y., Mier W. & Urban S. Fine mapping of pre-S sequence requirements for hepatitis B virus large envelope protein-mediated receptor interaction. J Virol 84, 1989–2000 (2010). 20007265 10.1128/JVI.01902-09 PMC2812397

[b36] Walter E., Keist R., Niederost B., Pult I. & Blum H. E. Hepatitis B virus infection of tupaia hepatocytes *in vitro* and *in vivo*. Hepatology 24, 1–5 (1996). 8707245 10.1002/hep.510240101

[b37] Galle P. R. . *In vitro* experimental infection of primary human hepatocytes with hepatitis B virus. Gastroenterology 106, 664–673 (1994). 8119538 10.1016/0016-5085(94)90700-5

[b38] Hennig B. J. . Host genetic factors and vaccine-induced immunity to hepatitis B virus infection. PLoS One 3, e1898 (2008). 18365030 10.1371/journal.pone.0001898 PMC2268746

[b39] Park B. L. . Association of common promoter polymorphisms of MCP1 with hepatitis B virus clearance. Exp Mol Med 38, 694–702 (2006). 17202846 10.1038/emm.2006.82

[b40] Zhou J. . Functional dissection of an IFN-alpha/beta receptor 1 promoter variant that confers higher risk to chronic hepatitis B virus infection. J Hepatol 51, 322–332 (2009). 19501422 10.1016/j.jhep.2009.03.020

[b41] Real C. I. . Hepatitis B virus genome replication triggers toll-like receptor 3-dependent interferon responses in the absence of hepatitis B surface antigen. Sci Rep 6, 24865 (2016). 27121087 10.1038/srep24865 PMC4848479

[b42] Sato S. . The RNA sensor RIG-I dually functions as an innate sensor and direct antiviral factor for hepatitis B virus. Immunity 42, 123–132 (2015). 25557055 10.1016/j.immuni.2014.12.016

[b43] Tzeng H. T. . Tumor necrosis factor-alpha induced by hepatitis B virus core mediating the immune response for hepatitis B viral clearance in mice model. PLoS One 9, e103008 (2014). 25047809 10.1371/journal.pone.0103008 PMC4105421

[b44] Ma Z., Zhang E., Yang D. & Lu M. Contribution of Toll-like receptors to the control of hepatitis B virus infection by initiating antiviral innate responses and promoting specific adaptive immune responses. Cell Mol Immunol 12, 273–282 (2015). 25418467 10.1038/cmi.2014.112 PMC4654312

[b45] Shlomai A. . Modeling host interactions with hepatitis B virus using primary and induced pluripotent stem cell-derived hepatocellular systems. Proc Natl Acad Sci USA 111, 12193–12198 (2014). 25092305 10.1073/pnas.1412631111 PMC4143014

[b46] Zhang R. R. . Efficient hepatic differentiation of human induced pluripotent stem cells in a three-dimensional microscale culture. Methods Mol Biol 1210, 131–141 (2014). 25173165 10.1007/978-1-4939-1435-7_10

[b47] Nagamoto Y. . The promotion of hepatic maturation of human pluripotent stem cells in 3D co-culture using type I collagen and Swiss 3T3 cell sheets. Biomaterials 33, 4526–4534 (2012). 22445253 10.1016/j.biomaterials.2012.03.011

[b48] Gieseck R. L. 3rd . Maturation of induced pluripotent stem cell derived hepatocytes by 3D-culture. PLoS One 9, e86372 (2014). 24466060 10.1371/journal.pone.0086372 PMC3899231

[b49] Baxter M. . Phenotypic and functional analyses show stem cell-derived hepatocyte-like cells better mimic fetal rather than adult hepatocytes. J Hepatol 62, 581–589 (2015). 25457200 10.1016/j.jhep.2014.10.016 PMC4334496

[b50] Wu X. . Productive hepatitis C virus infection of stem cell-derived hepatocytes reveals a critical transition to viral permissiveness during differentiation. PLoS Pathog 8, e1002617 (2012). 22496645 10.1371/journal.ppat.1002617 PMC3320597

[b51] Mizuguchi H. & Kay M. A. Efficient construction of a recombinant adenovirus vector by an improved *in vitro* ligation method. Hum Gene Ther 9, 2577–2583 (1998). 9853524 10.1089/hum.1998.9.17-2577

[b52] Mizuguchi H. & Kay M. A. A simple method for constructing E1- and E1/E4-deleted recombinant adenoviral vectors. Hum Gene Ther 10, 2013–2017 (1999). 10466635 10.1089/10430349950017374

[b53] Sugiyama M. . Influence of hepatitis B virus genotypes on the intra- and extracellular expression of viral DNA and antigens. Hepatology 44, 915–924 (2006). 17006908 10.1002/hep.21345

[b54] Murakami S. . Interaction of penton base Arg-Gly-Asp motifs with integrins is crucial for adenovirus serotype 35 vector transduction in human hematopoietic cells. Gene Ther 14, 1525–1533 (2007). 17805302 10.1038/sj.gt.3303019

[b55] Maizel J. V. Jr., White D. O. & Scharff M. D. The polypeptides of adenovirus. I. Evidence for multiple protein components in the virion and a comparison of types 2, 7A, and 12. Virology 36, 115–125 (1968). 5669982 10.1016/0042-6822(68)90121-9

[b56] Watashi K. . Cyclosporin A and its analogs inhibit hepatitis B virus entry into cultured hepatocytes through targeting a membrane transporter, sodium taurocholate cotransporting polypeptide (NTCP). Hepatology 59, 1726–1737 (2014). 24375637 10.1002/hep.26982 PMC4265264

