# Supplementary material for: Human induced-pluripotent stem cell-derived hepatocyte-like cells as an in vitro model of human hepatitis B virus infection
Source: Sci Rep. 2017 Apr 4;7:45698. doi: 10.1038/srep45698 (PMC5379564; doi:10.1038/srep45698)
Supplement: Supplementary Information [file srep45698-s1.pdf]

Supplemental information

Title

Human induced-pluripotent stem cell-derived hepatocyte-like cells as an *in vitro* model of human hepatitis B virus infection

Author Information

Fuminori Sakurai, Seiji Mitani, Tatsuro Yamamoto, Kazuo Takayama, Masashi Tachibana, Koichi Watashi, Takaji Wakita, Sayuki Iijima, Yasuhito Tanaka, Hiroyuki Mizuguchi

Supplemental Figure 1

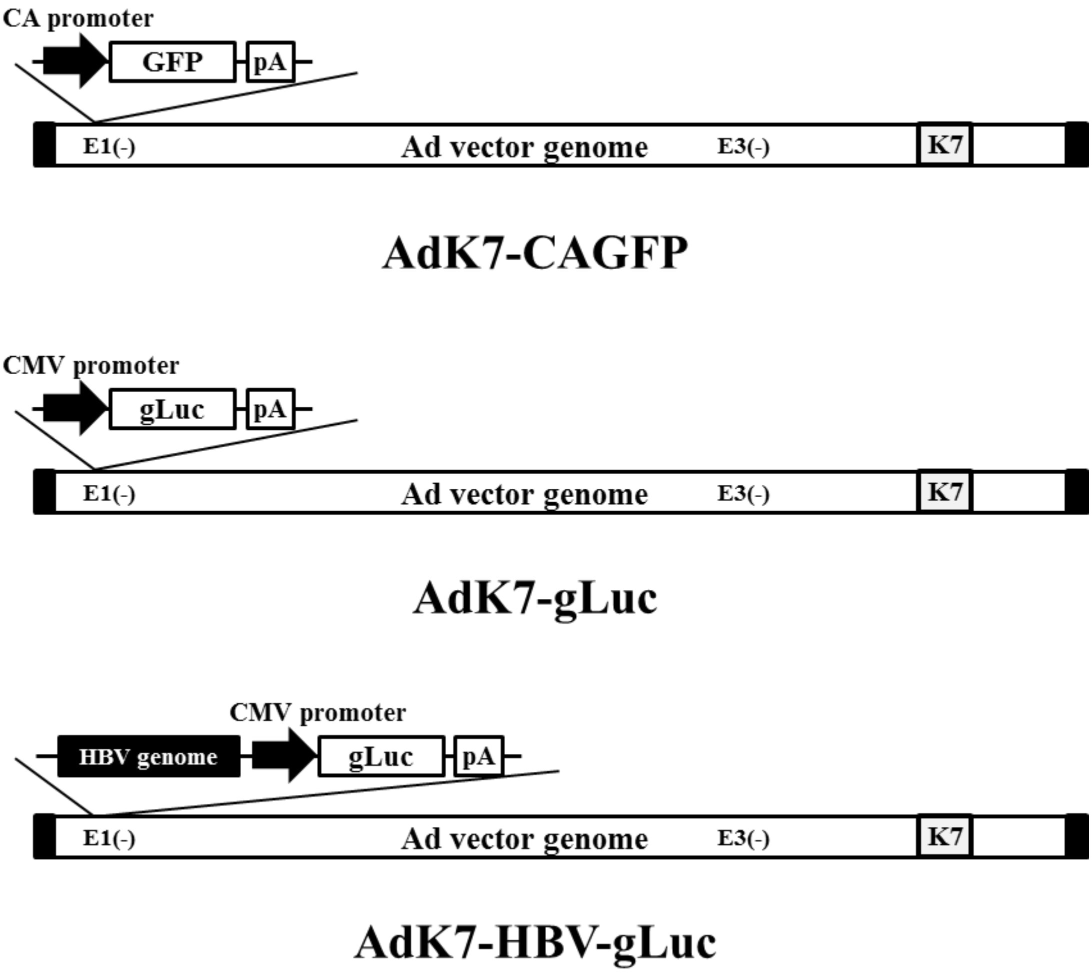

Supplemental Fig. 1. Schematic diagram of Ad vectors used in this study. CA promoter: a hybrid promoter composed of chicken beta-actin promoter and cytomegalovirus (CMV) early enhancer; gLuc: gaussia luciferase; pA: bovine growth hormone polyA signal; K7: polylysine-coding sequence.

Supplemental Figure 2

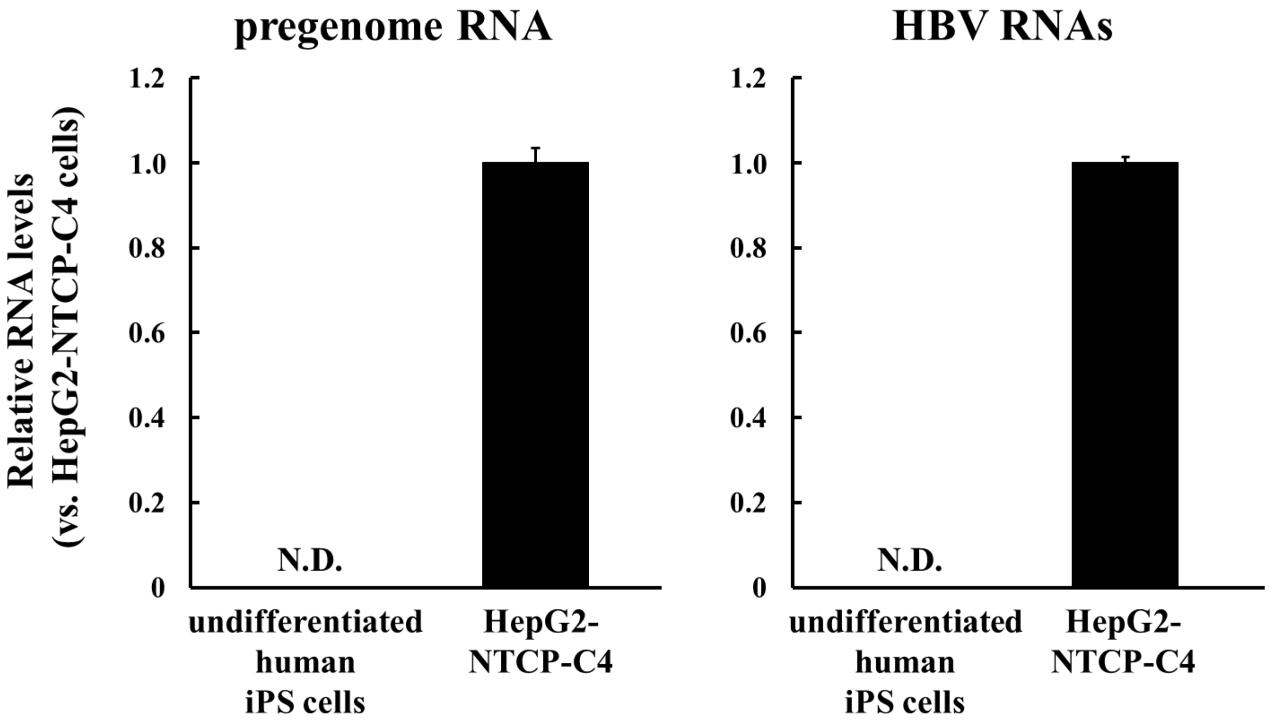

Supplemental Fig. 2. Levels of pgRNA and HBV RNAs following inoculation with HBV in human iPS cells. Human iPS cells and HepG2-NTCP-C4 cells were inoculated with HBV (genotype D) at 5000 GEq/cell. Following a 24-h incubation, cells were gently washed, followed by addition of fresh medium. Following a total 120-h incubation, total RNA was recovered from the cells, followed by real-time RT-PCR analysis. The ratios of pgRNA and HBV RNAs to GAPDH levels were determined. The ratio of HepG2-NTCP-C4 cells was normalized to 1. The data are presented as the mean ± S.D. (n=3). N.D.: not detected.

Supplemental Figure 3

a

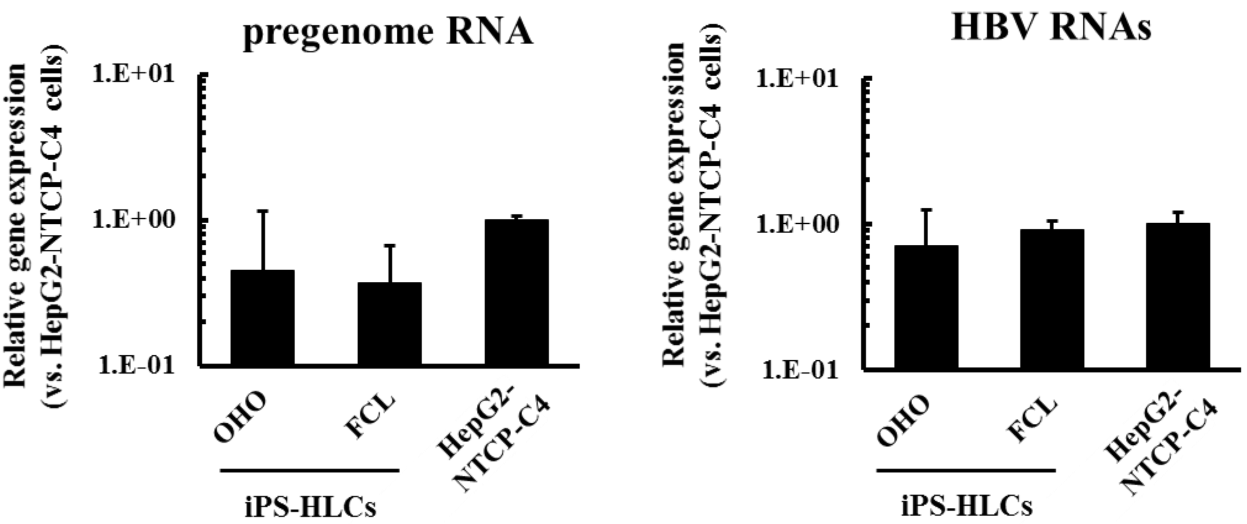

b

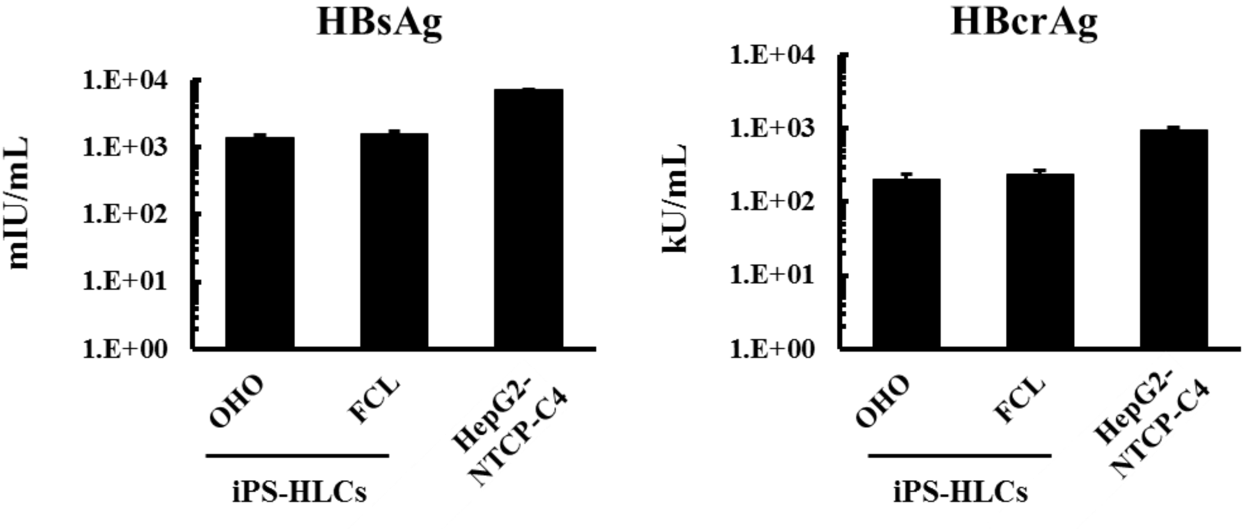

Supplemental Fig. 3. HBV infection in iPS-HLCs differentiated from the other human iPS cell lines. (a) Expression levels of pgRNA and HBV RNAs following inoculation with HBV in iPS-HLCs. (b) Expression levels of HBsAg and HBcrAg in the culture supernatants following inoculation with

HBV in iPS-HLCs. iPS-HLCs and HepG2-NTCP-C4 cells were inoculated with HBV (genotype D) at 5000 GEq/cell for 24 h. Expression levels of pgRNA and HBV RNAs were determined by real-time RT-PCR analysis 10 days after inoculation. The ratios of pgRNA and HBV RNAs to GAPDH mRNA levels were determined. The ratio of HepG2-NTCP-C4 cells was normalized to 1. HBV protein levels in the culture supernatants were determined 10 days after inoculation by CLEIA. The data are presented as the mean ± S.D. (n=3).
